# Stevens–Johnson syndrome-toxic epidermal necrolysis overlap in a patient taking quetiapine and famotidine: a case report

**DOI:** 10.1186/s13256-024-04629-6

**Published:** 2024-07-28

**Authors:** Chi-Sheng Su, Chi-Lan Kao

**Affiliations:** Department of Pharmacy, Cathay General Hospital Medical Center, No 280, Sec 4, Renai Road, Taipei, 10630 Taiwan, Republic of China

**Keywords:** Stevens–Johnson syndrome, Quetiapine fumarate, Antipsychotic agents, Famotidine, Histamine H2 antagonists, Case reports

## Abstract

**Background:**

Stevens–Johnson syndrome-toxic epidermal necrolysis (SJS-TNE) overlap is a rare skin disorder characterized by erythema, blisters, extensive exfoliation, epidermal detachment, the involvement of multiple mucosae, and positive Nikolsky’s sign. SJS-TEN has a high mortality rate. Our case involves a rare occurrence of drug-induced Stevens–Johnson syndrome-toxic epidermal necrolysis overlap with a delayed onset in the setting of quetiapine and famotidine therapy.

**Case presentation:**

An 82-year-old Taiwanese female was admitted to our hospital for decreased urine output, generalized edema, and multiple skin blisters and bedsores. With further spread of the lesions, multiple ruptured bullae with shallow erosions on the face, trunk, and limbs and mucosal involvement affected 20% of the total body surface area. Nikolsky’s sign was positive. A diagnosis of Steven–Johnson syndrome was highly suspected. One month prior, she had started famotidine and quetiapine. Intravenous methylprednisolone treatment was initiated, which ameliorated the skin lesions after 3 days. However, new lesions developed after only 1 day of methylprednisolone tapering. The patient died 12 days after admission.

**Conclusion:**

Stevens–Johnson syndrome-toxic epidermal necrolysis is a rare skin disorder. Although it is mainly acute and has a high mortality rate, delayed onset can still occur. Quetiapine and famotidine are generally safe and effective for treating geriatric and gastrointestinal problems, but rare drug hypersensitivity reactions can lead to debilitating consequences. Therefore, increased clinical awareness and the initiation of supportive care are imperative. Optimal management guidelines are still lacking, and confirmation of developed guidelines through randomized controlled trials is needed. Collaboration for better management strategies is warranted.

## Background

Stevens-Johnson syndrome (SJS) and toxic epidermal necrolysis (TEN) are rare and potentially fatal mucocutaneous reactions characterized by blisters and skin exfoliation. Drugs and infections are the leading causes.

SJS and TEN are in the same spectrum of diseases but affect different total body surface area percentages [1]: SJS, < 10%; SJS–TEN overlap, 10–30%; and TEN, > 30%. The overall incidence of SJS–TEN is unclear, although a US study indicated that the incidence could be between 1.58 and 2.26 per million people annually, and the mortality rates could be as high as 4.8–14.8% [2]. Even after recovery, blindness can be a serious sequela in some patients. Thus, prompt diagnosis and appropriate treatment are necessary.

From the SCAR study, a case‒control study that evaluated the risks of SJS–TEN for patients taking various medications, and medications associated with high relative risks were cotrimoxazole, carbamazepine, phenytoin, phenobarbital, NSAIDs of the oxicam type, allopurinol, chlormezanone, aminopenicillins, cephalosporins, quinolones, and cyclic antibiotics [3]. Among antipsychotic agents, clozapine, aripiprazole, quetiapine, and ziprasidone have been associated with SJS–TEN [4, 5], but thus far, no case reports for quetiapine and ziprasidone have been published. Among the histamine H2 antagonists, both famotidine and ranitidine have been associated with SJS–TEN [6], and famotidine was described in only one case report.

Herein, we present the case of a patient with a drug reaction leading to Stevens–Johnson syndrome-toxic epidermal necrolysis (SJS–TEN) overlap with a delayed onset in the setting of quetiapine and famotidine therapy.

## Case presentation

An 82-year-old Taiwanese female was admitted to our hospital for decreased urine output, generalized edema, multiple skin blisters, and bedsores. The patient had a history of hypertension, chronic kidney disease, severe anemia, and gouty arthropathy. She had dementia since 70 years of age and had been bedridden for several years. Before her admission, she was under hospice home care. She received the following medications regularly: amlodipine for hypertension (well controlled), acetaminophen for pain control, and folic acid for anemia. In the previous 1.5 months, the following drugs were added: methoxy polyethylene glycol-epoetin beta and ferrous citrate for anemia, sodium bicarbonate for chronic kidney disease, famotidine for gastrointestinal (GI) discomfort, quetiapine for mental health issues, and tramadol/acetaminophen for additional pain control. She had no known drug allergies.

In the emergency room, her wounds were washed with chlorhexidine solution, and an antibiotic ointment containing bacitracin, neomycin, and polymyxin B was applied.

On admission, the patient had no fever, with a body temperature of 35.9 °C, a heart rate of 76 beats/minute, a respiratory rate of 20 breaths/minute, and a blood pressure of 116/59 mmHg. The patient was cachectic and malnourished, and enteral nutrition caused her to produce tarry stool. Furosemide was initiated for her generalized edema, fusidic acid cream for her blisters and silver sulfadiazine for her bedsores.

Laboratory studies revealed a hemoglobin level of 7.8 g/dL (reference range = 12–16 g/dL), a sodium level of 120 mmol/L (reference range, 135–145 mmol/L), a blood urea nitrogen level of 50 mg/dL (reference range, 8–25 mg/dL), a serum creatinine level of 4.03 mg/dL (reference range, 0.44–1 mg/dL), a white blood cell count of 9.54 × 1000/μL (reference range, 4–10 × 1000/μL), 92% neutrophils (reference range, 40–75%), 1.0% lymphocytes (reference range, 20–45%), and 7.0% eosinophils (reference range, 0–7.0%).

**Fig. 1:**
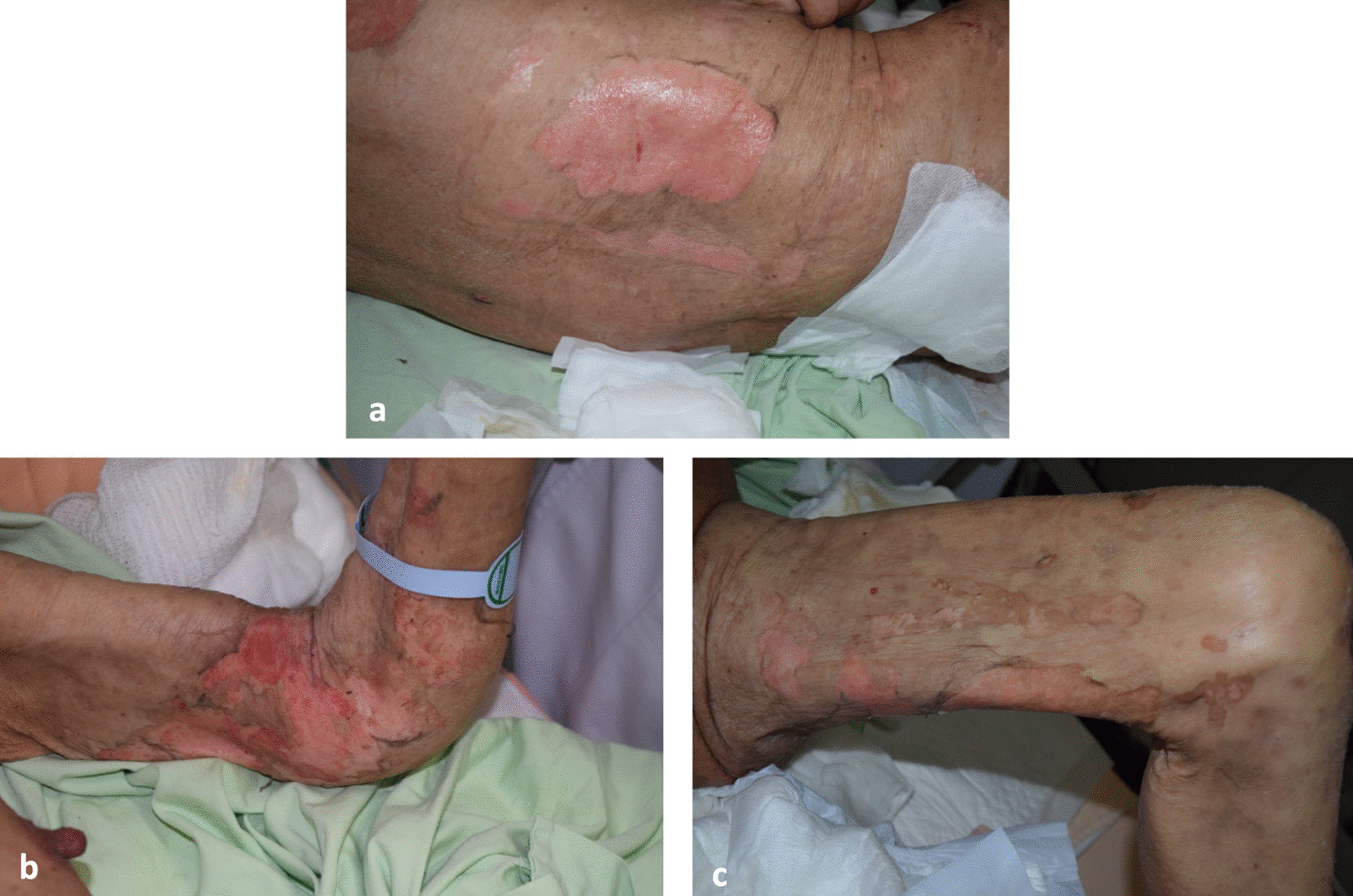
Epidermal detachment with exposed dermis. **a** Lower back, **b** upper limb, and **c** thigh

**Fig. 2:**
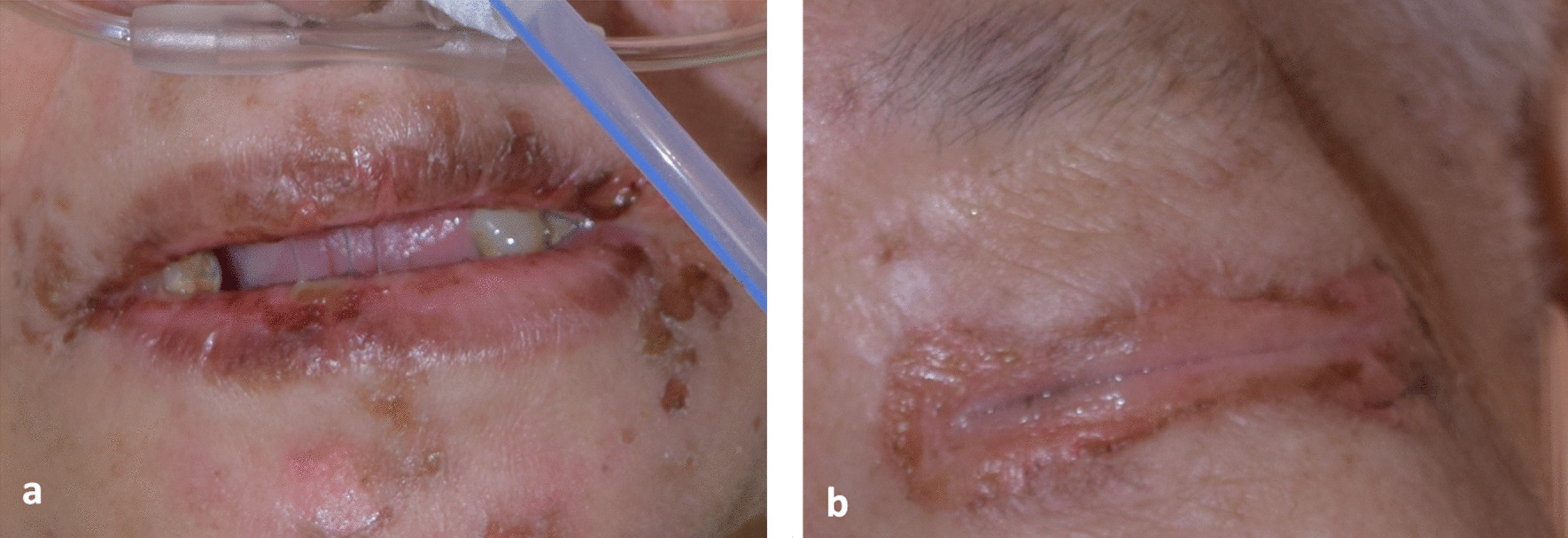
Mucosal sites affected by Stevens–Johnson syndrome-toxic epidermal necrolysis. **a** Mouth and **b** eyes

We consulted a dermatologist on hospital day 3 due to the presence of many vesicles with skin and oral involvement. Cutaneous examination by our dermatologist revealed multiple ruptured bullae with shallow erosions on the face, trunk, and limbs, affecting 20% of the total body surface area (TBSA). Nikolsky’s sign was positive. Her oral, genital, and ocular mucosa were also involved (Figs. 1 and 2). A diagnosis of Steven–Johnson syndrome was highly suspected. Her family refused skin biopsy, and a do-not-resuscitate consent form had been signed. Treatment with intravenous methylprednisolone (40 mg, every 8 h) was initiated. Quetiapine, tramadol/acetaminophen, and silver sulfadiazine were replaced with haloperidol, morphine, and neomycin ointment. The last doses of methoxy polyethylene glycol-epoetin beta and ferrous citrate had been administered more than 1 month before the skin reaction, and the patient had used tramadol/acetaminophen many times over the several years prior to presentation at our hospital.

On hospital day 5, multiple new skin abrasions with green-colored discharge were noted, and an empirical antibiotic of piperacillin/tazobactam was initiated. The culture results on a pus sample obtained during the patient’s hospitalization were positive for *Pseudomonas aeruginosa*, group G β-hemolytic *Streptococcus*, *Proteus mirabilis*, and *Bacteroides thetaiotaomicron*. Therefore, antibiotic treatment with piperacillin/tazobactam was continued.

Her skin lesions resolved after 3 days of treatment with methylprednisolone; thus, the dose of methylprednisolone was tapered to 40 mg every 12 hours. However, new skin lesions developed 1 day after methylprednisolone tapering. Later, coffee-ground emesis was noted; thus, famotidine was replaced with intravenous pantoprazole. At this point, quetiapine and famotidine were the last two suspected medications to be withdrawn during the course of hospitalization.

After 12 days in the hospital, the patient had hypothermia and hypotension. Although fluid resuscitation and vasopressor use were suggested, treatment was refused by her family. The patient died 12 days after admission.

Other medications also used during the course of treatment included acetylcysteine, chlorpheniramine, morphine, dexamethasone oral paste, tetracycline ointment, and antibiotic ointment, which included nystatin, neomycin, gramicidin, and triamcinolone acetonide.

## Discussion and conclusions

SJS–TEN is a rare skin disorder that affects 1.58–2.26 per million people annually. The mortality rate can be as high as 4.8–14.8% [2]. Although a drug reaction (mostly dose independent) is the common cause, cases of SJS–TEN caused by infections have been documented.

The pathogenesis of SJS–TEN is not fully understood. Keratinocyte apoptosis is believed to be the cause of widespread epidermal cell death. Recent research has demonstrated that the blistered and inflamed skin of patients with SJS–TEN is extensively infiltrated by effector memory polycytotoxic CD8^+^ T cells, and these cells are drug specific [7, 8].

The clinical course of SJS–TEN is characterized by an incubation period of approximately 1–3 weeks, and it is not uncommon for SJS–TEN to progress even after causal medication withdrawal for days or even weeks. Three phases of SJS–TEN have been suggested: a prodromal phase, acute phase, and recovery phase. Fever and upper respiratory tract infection-like symptoms are common in the prodromal phase. Some patients may also experience conjunctivitis, sore throat, generalized erythema, and difficulty in urination. In the acute phase, which often lasts 8–12 days, patients may experience fever, generalized sloughing, and mucus membrane symptoms. Cutaneous areas demonstrate Nikolsky’s sign. Mucositis and stomatitis cause intake reduction, malnutrition, and dehydration. Urethritis may cause urinary retention. In the recovery phase, cutaneous symptoms may persist for 2 weeks, and mucus involvement may persist for 2 months. Patients with mucosal involvement are more vulnerable to infection, which is associated with a poor prognosis. Leucopenia and thrombocytopenia may progress within a few days, and septic complications may ensue, leading to multiorgan failure and death [9]. The symptoms mentioned above were typical for our patient, except she had no fever. And the onset was more than 3 weeks.

The Severity of Illness Score of Toxic Epidermal Necrolysis (SCORTEN) is based upon seven independent clinical and laboratory variables and can be used to predict the risk of mortality for a patient. Because our patient was receiving home hospice care, some laboratory test results were not available from that time. With the clinical and laboratory results that were available, the SCORTEN would have been three points, which indicates a mortality risk of 35.3% [10].

Although the typical treatment for SJS–TEN in the clinical setting is systemic corticosteroids, few studies have shown the benefit of corticosteroid therapy over supportive care. In a recent meta-analysis that included 96 studies (3248 patients) conducted between 1990 and 2012, Zimmermann *et al*. demonstrated that the results of only three studies of corticosteroid therapy suggested a benefit, and the differences of only one of the three studies were statistically significant. All the other treatments in this study failed to show survival benefits over supportive care. The treatments included in this study were intravenous immunoglobulins, cyclosporine, plasmapheresis, thalidomide, cyclophosphamide, hemoperfusion, tumor necrosis factor inhibitors, and granulocyte colony-stimulating factors [11]. A beneficial effect of corticosteroids might exist when specific treatment modalities are applied, such as early administration (within 7 days) or pulse therapy (short-term high dose), or within selected subgroups [11]

The findings of another SCORTEN-based systematic review and meta-analysis conducted by Torres-Navarro *et al*. suggested that cyclosporine and immunoglobulins plus corticosteroids were associated with fewer deaths than predicted by SCORTEN. However, in the network meta-analysis, no treatment significantly reduced the standardized mortality ratio (SMR), the quotient of the observed and SCORTEN-predicted mortality [12]. In a recent Japanese retrospective study conducted by Watanabe *et al*., 132 patients with SJS and TEN from two university hospitals were included. The mortality rates between 2000 and 2019 for SJS and TEN were 1.3% and 12.5%, respectively. However, between 2012 and 2019, the mortality rate for patients with TEN was 3.8%. The author suggested that pulse steroid therapy combined with plasma exchange and/or immunoglobulin therapy improved the outcomes of patients with TEN compared with systemic steroid therapy alone; although, the difference was not statistically significant [13]. Our patient was treated with systemic corticosteroids. Although the symptoms improved within 3 days of treatment, new skin lesions appeared after 1 day of corticosteroid tapering.

In a previous case report for famotidine, the onset of SJS–TEN was 5 days after famotidine initiation [6]. Another study by Rzany *et al*. [14] reported that for most patients, the onset of skin symptoms occurred approximately 2 weeks after the administration of H2-blockers, including famotidine. For our patient, the onset of SJS–TEN occurred approximately 4 weeks after famotidine initiation. Although there was a relative delay in the onset of SJS–TEN, causality cannot be ruled out.

Cases of SJS–TEN related to quetiapine have been documented for many years, but there are few case reports describing the clinical course of SJS–TEN caused by this medication. To diagnose drug-related SJS–TEN, it is suggested that medications initiated within 24 hours and beyond 3 weeks be ruled out [9]. This suggestion might partly explain why there are few case reports that describe quetiapine-related SJS–TEN. However, phenytoin-induced TEN is an exception, as it may develop 2–8 weeks after phenytoin initiation [9]. Quetiapine-induced STS–TEN might be another exception with a relatively delayed onset. For our patient, the onset of SJS–TEN occurred approximately 4 weeks after quetiapine initiation.

In patients with chronic kidney disease, the clearance of quetiapine is 25% less than that in patients with normal renal function [15]. As a result, the accumulation of quetiapine in our patient could be one of the causes of the recurrence of SJS–TEN. Another causal medication could be famotidine, which was not withdrawn at the onset of SJS–TEN. Corticosteroids could have masked the symptoms caused by one or both medications, with corticosteroid tapering leading to recurrence. We do not know whether the recurrence was due to tapering corticosteroids too early or other causes. The patient’s condition deteriorated swiftly, and she died 12 days after admission.

Further research on the infiltrated T cells in SJS–TEN blisters might elucidate the pathogenesis of this rare skin disorder. With the advancement of whole-genome sequencing, the avoidance of medication use in patients harboring susceptibility genes might be possible. The lack of optimal management guidelines calls for their development and confirmation through randomized controlled trials.

SJS–TEN is a rare skin disorder. Although the disease is primarily acute and has a high mortality rate, delayed onset can still occur. This delay makes diagnosis extremely challenging. Quetiapine and famotidine are generally safe and effective for treating geriatric and gastrointestinal problems, but rare drug hypersensitivity reactions can lead to debilitating consequences. Therefore, increased clinical awareness and the initiation of supportive care are imperative. Optimal management guidelines are still lacking, and confirmation of developed guidelines through randomized controlled trials is needed. Collaboration for better management strategies is warranted.

## Data Availability

Data sharing is not applicable to this article, as no datasets were generated or analyzed during the current study.

## Abbreviations

TEN: Toxic epidermal necrolysis
SJS: Stevens–Johnson syndrome
